# Dynamic Assessment of Modified EASIX (m-EASIX) at 48 Hours Predicts Adverse Outcomes in Acute Pancreatitis: A Propensity Score-Matched Study

**DOI:** 10.3390/medicina62030568

**Published:** 2026-03-18

**Authors:** Hikmet Öztop, Enes Yavuz, Nevriye Gül Ada Tak, Fatih Eren, Fazıl Çağrı Hunutlu

**Affiliations:** 1 Department of Internal Medicine, Faculty of Medicine, Bursa Uludag University, Bursa 16059, Turkey; enesyavuz95@gmail.com (E.Y.); ngulada@gmail.com (N.G.A.T.); 2 Division of Gastroenterology, Department of Internal Medicine, Faculty of Medicine, Bursa Uludag University, Bursa 16059, Turkey; fatiheren@uludag.edu.tr; 3 Division of Hematology, Department of Internal Medicine, University of Health Sciences, Bursa Sehir Training and Research Hospital, Bursa 16110, Turkey; fazilhunutlu@gmail.com

**Keywords:** acute pancreatitis, m-EASIX, endothelial dysfunction, risk stratification, clinical outcomes

## Abstract

*Background and Objectives*: Early risk stratification in acute pancreatitis (AP) remains challenging, particularly for identifying patients who initially appear low-risk but later develop complications. The Modified Endothelial Activation and Stress Index (m-EASIX) reflects endothelial injury and systemic inflammation. This study evaluated the prognostic value of dynamic 48 h m-EASIX assessment for predicting adverse clinical outcomes in AP. *Materials and Methods:* This retrospective cohort study included adult patients hospitalized with AP between January 2020 and June 2025. Propensity score matching (1:1) was performed using age, sex, BISAP score and etiology. Laboratory parameters were recorded at admission and at 48 h. Adverse outcomes were defined as prolonged hospitalization (≥8 days) and/or pancreatic necrosis, abscess, intensive care unit admission or in-hospital mortality. Multivariable logistic regression was used to identify independent predictors of adverse outcomes. Receiver operating characteristic (ROC) analysis evaluated the predictive performance of m-EASIX and compared it with BISAP and Ranson scores. *Results:* A total of 258 patients were included in the initial cohort, of whom 93 experienced an adverse clinical course. After propensity score matching, 170 patients remained in the final analysis (85 per group). The 48 h m-EASIX score was independently associated with adverse outcomes in both unmatched and matched cohorts. ROC analysis showed a moderate discrimination for composite outcomes (AUC ≈ 0.76) and a stronger discrimination for hard outcomes (AUC up to 0.867). In all analyses, m-EASIX significantly outperformed BISAP and Ranson scores (DeLong test *p* < 0.001). Dynamic risk reclassification showed that m-EASIX identified a subgroup of patients initially classified as low-risk by BISAP who later developed adverse outcomes. *Conclusions:* The dynamic assessment of m-EASIX at 48 h provides additional prognostic information for early risk stratification in AP and may help identify patients at an increased risk of unfavorable clinical courses.

## 1. Introduction

Acute pancreatitis (AP) is an inflammatory condition that develops as a result of the inappropriate activation of pancreatic enzymes due to various etiological factors [1]. The diagnosis of AP is based on the 2012 revised Atlanta criteria and is established by the presence of at least two of the following findings: characteristic abdominal pain; serum lipase or amylase levels greater than three times the upper limit of normal; and characteristic findings on computed tomography (CT) or magnetic resonance imaging (MRI) [2].

Most patients with AP experience a mild disease course with symptoms usually resolving within one week. However, approximately 20% of patients develop moderately severe or severe AP (SAP), characterized by pancreatic necrosis and even organ failure [3,4]. In the PANC Study, the mean length of hospital stay was reported as 4 days in patients with mild AP, 8 days in those with moderately severe disease and 17 days in patients with severe disease [5].

Various clinical and laboratory-based scoring systems have been proposed to evaluate disease severity and clinical outcomes in AP. First described in 1974, the Ranson score was the earliest scoring system used to predict prognosis; however, despite its widespread use, its clinical applicability is limited by the large number of parameters it requires [6]. The Bedside Index for Severity in Acute Pancreatitis (BISAP) is a widely used alternative [7]. In comparison to the Ranson score, BISAP has practical advantages because it can be utilized upon hospital admission and depends on fewer parameters [8]. While the existing scoring systems accurately identify mild cases, their ability to predict moderately severe to severe AP—associated with complications and prolonged hospital stays—remains limited [9,10]. Current international guidelines highlight the need to evaluate dynamic changes during the disease course in managing AP [11].

Endothelial dysfunction plays a critical role in the pathophysiology of AP and contributes to both local and systemic tissue injury through inflammatory and microcirculatory mechanisms [12,13]. The Endothelial Activation and Stress Index (EASIX) is a scoring tool used to assess endothelial injury and the associated inflammatory response [14]. Initial studies investigating the EASIX score primarily focused on acute graft-versus-host disease (aGVHD), a severe complication of allogeneic hematopoietic stem cell transplantation characterized by significant endothelial injury. These studies demonstrated that higher EASIX scores were associated with an increased risk of developing aGVHD [15]. The modified EASIX score (m-EASIX) was subsequently developed by replacing the creatinine parameter in the standard EASIX score with C-reactive protein (CRP) and has been proposed as a marker reflecting the severity of inflammation and endothelial injury [16]. In subsequent studies, EASIX and m-EASIX scores have been reported to be useful in predicting clinical outcomes in various malignancies and inflammatory clinical conditions associated with endothelial dysfunction [17,18,19,20,21,22].

However, there is currently no available evidence regarding the clinical utility of m-EASIX in patients with AP. The 48 h time point was selected because early dynamic changes during the first 48 h are considered critical in the clinical course of acute pancreatitis. In routine clinical practice, reassessment at approximately 48 h is commonly used to evaluate disease progression, guide imaging decisions and refine risk stratification, as reflected in established scoring systems such as the Ranson score. Therefore, evaluating m-EASIX at this time point may provide clinically relevant prognostic information beyond admission parameters.

The objective of this study was to assess whether the m-EASIX score, which utilizes commonly available laboratory parameters and dynamic variables, can effectively predict adverse clinical events, such as complications or an extended hospital stay, during the early stages of AP.

## 2. Materials and Methods

### 2.1. Study Design and Population

This retrospective cohort study included patients hospitalized with an initial diagnosis of acute pancreatitis (AP) at a tertiary care center from January 2020 to June 2025. Clinical, laboratory and radiological data were obtained from electronic medical records.

### 2.2. Inclusion and Exclusion Criteria

Adult patients aged ≥18 years with a diagnosis of AP were eligible for inclusion. The diagnosis was confirmed using standard diagnostic criteria [2].

**Figure 1:**
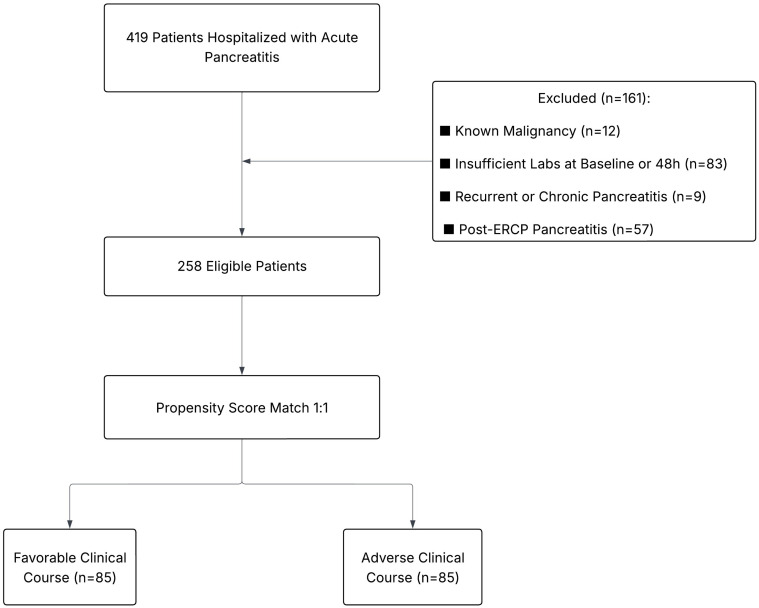
Flowchart illustrating patient selection, exclusion criteria and propensity score matching.

Patients were excluded from the study if they met any of the following criteria: a known history of malignancy; inadequate laboratory or radiological data at the time of diagnosis; a history of recurrent pancreatitis; a confirmed diagnosis of chronic pancreatitis; pancreatitis following endoscopic retrograde cholangiopancreatography (ERCP); or insufficient laboratory data available at the 48 h follow-up (Figure 1).

### 2.3. Clinical Course Classification and Outcomes

Patients were categorized into two groups based on their clinical course: favorable and adverse. A favorable clinical course was defined as a hospital stay lasting ≤ 7 days. An adverse course was defined as hospitalization ≥ 8 days and/or the development of complications, including pancreatic necrosis, abscess formation, ICU admission or in-hospital mortality. This composite outcome reflects clinically significant disease severity, extending beyond just the duration of hospitalization. None of the patients experienced prolonged hospitalization due to same-admission cholecystectomy, as the procedure was performed electively after clinical stabilization and discharge.

### 2.4. Clinical Management and Discharge Criteria

All patients received standard care management according to contemporary guidelines. This included early intravenous fluid resuscitation, adequate pain relief, antiemetic therapy and the early initiation of oral or enteral nutrition as tolerated. Clinical monitoring consisted of regular assessments of vital signs, laboratory parameters and imaging when clinically necessary. Imaging studies were conducted following standard clinical practices and current guidelines for managing acute pancreatitis. Contrast-enhanced computed tomography (CECT) was not routinely performed upon admission but was usually obtained after 48 to 72 h if clinically necessary. Indications for imaging included persistent or worsening abdominal pain, clinical deterioration, lack of expected improvement, rising inflammatory markers or suspicion of local complications. Additional imaging was also carried out as needed to assist with clinical management.

Pancreatic necrosis was defined as areas of non-enhancing pancreatic tissue or peripancreatic tissue observed on contrast-enhanced CT. A pancreatic abscess was diagnosed when there was a well-defined fluid collection with imaging features indicative of infection, as documented in the radiology reports. Discharge decisions were based on criteria such as clinical stability, effective pain control with oral analgesics, the ability to tolerate oral feeding, the absence of uncontrolled complications and hemodynamic stability.

### 2.5. Severity Scores and Risk Stratification

BISAP scores were assessed at admission and categorized as low-risk (0–2) or high-risk (≥3). Ranson scores were calculated using parameters from both admission and 48 h later, categorized as mild (0–2) or severe (≥3) [23].

### 2.6. Laboratory Measurements and Derived Indices

Laboratory parameters were documented at admission (0 h) and at 48 h, including complete blood count parameters, platelet count, lactate dehydrogenase (LDH), creatinine and C-reactive protein (CRP). Endothelial and inflammation-based indices were calculated using established formulas, including EASIX and m-EASIX. The m-EASIX was calculated using the formula (LDH × CRP)/platelet count, where LDH was expressed in U/L, CRP in mg/L and platelet count in ×10^9^/L. No scaling or logarithmic transformation was applied to the individual laboratory variables before index calculation. Additional indices of inflammation, including the neutrophil-to-lymphocyte ratio (NLR), platelet-to-lymphocyte ratio (PLR), systemic immune inflammation index (SII), systemic inflammation response index (SIRI) and pan-immune inflammation value (PIV), were calculated according to previously established definitions [24,25]. The change in m-EASIX between admission and 48 h (Δm-EASIX) was also calculated.

### 2.7. Propensity Score Matching

Propensity score matching (PSM) was performed using Python (version 3.13, Anaconda distribution) alongside the pandas, NumPy and scikit-learn libraries. Propensity scores were estimated using logistic regression, incorporating the following baseline covariates: age, sex, BISAP score and etiology (biliary vs. non-biliary). Patients were matched in a 1:1 ratio using nearest-neighbor matching without replacement and without a caliper, based on the absolute differences in their propensity scores.

Covariate balance was assessed using standardized mean differences (SMDs) before and after matching, and the results were visualized with a Love plot. An absolute SMD of less than 0.10 was deemed indicative of adequate balance. Matching without a caliper restriction was preferred to preserve sample size while maintaining acceptable covariate balance. All subsequent analyses were performed on the matched cohort.

### 2.8. Statistical Analysis

Statistical analyses and data visualization were conducted using SPSS version 29.0 (IBM Corp., Armonk, NY, USA) and R version 4.5.2 (R Foundation for Statistical Computing, Vienna, Austria) within RStudio (Posit Software, Boston, MA, USA). Data management was performed using the tidyverse and haven packages. Continuous variables are presented as mean ± standard deviation or median (interquartile range), as appropriate. Categorical variables were presented as frequencies and percentages.

In the unmatched cohort, comparisons between groups were performed using the independent samples *t*-test or Mann–Whitney U test for continuous variables and the Pearson chi-square test or Fisher’s exact test for categorical variables.

For the matched cohort, paired statistical tests were used. Continuous variables were analyzed using the paired *t*-test or Wilcoxon signed-rank test and categorical variables were analyzed using McNemar’s test.

Logistic regression analysis was used to identify predictors of adverse clinical course. In the matched cohort, logistic regression models with robust standard errors clustered by matched pair were applied.

Receiver operating characteristic (ROC) curve analysis was used to evaluate the predictive performance of m-EASIX, BISAP, and Ranson scores. The Youden index determined the optimal cut-off value. Area under the curve (AUC) values were calculated and compared using the DeLong test with the pROC package.

The longitudinal trajectory of risk stratification from baseline (BISAP) to the 48 h assessment (Ranson and m-EASIX) and its association with adverse outcomes was visualized using Sankey diagrams via the ggalluvial package.

Univariate logistic regression analysis was conducted to identify variables associated with an adverse clinical course. Variables with *p* < 0.25 in univariate analysis were considered for entry into the multivariable logistic regression model. Multicollinearity among candidate variables was evaluated using the variance inflation factor (VIF), and only variables with VIF values < 2 were retained. Backward elimination based on the likelihood ratio (LR) test was applied to obtain the final model, and variables with *p*-values < 0.05 were considered statistically significant. Model calibration of the primary multivariable logistic regression model was assessed using the Hosmer–Lemeshow goodness-of-fit test.

A sensitivity analysis was performed to assess the robustness of the primary outcome definition. In this analysis, only hard clinical outcomes were taken into account, including pancreatic necrosis, abscess, admissions to the intensive care unit and in-hospital mortality. Length of hospital stay was excluded from the endpoints. The predictive performance of the m-EASIX (48 h) score was then re-evaluated using ROC curve analysis and was compared with the BISAP and Ranson scores in both matched and unmatched cohorts.

The clinical utility of the m-EASIX (48 h) score was evaluated using decision curve analysis (DCA) to determine its net benefit in predicting outcome compared to BISAP and Ranson scores. For the DCA, raw scores were first converted into predicted probabilities using logistic regression models. Net benefit was then calculated across a spectrum of threshold probabilities and compared against two baseline strategies: ‘treat-all’ (assuming all patients have the outcome) and ‘treat-none’ (assuming no patients have the outcome). This analysis was performed for both the unmatched and PSM-matched cohorts to ensure the robustness of the findings. Improvement in risk classification was evaluated using continuous net reclassification improvement (NRI) and integrated discrimination improvement (IDI). NRI was calculated based on changes in predicted risks for events and non-events, and IDI was computed as the difference in discrimination slopes between models.

To assess statistical uncertainty, bootstrap resampling with 2000 iterations was applied to estimate 95% confidence intervals for NRI and IDI. Analyses were performed in both PSM and unmatched cohorts to evaluate the robustness of the findings.

## 3. Results

### 3.1. Patient Characteristics and Balance of Covariates Following Propensity Score Matching

A total of 258 patients were included in the initial cohort, comprising 165 patients with a favorable clinical course and 93 with an adverse clinical course. After 1:1 nearest-neighbor matching without replacement, a total of 170 patients were included in the final analysis, with 85 patients in each group: favorable and adverse clinical courses. Baseline demographic, clinical and laboratory characteristics of the unmatched and matched cohorts are presented in Table 1. An adequate covariate balance was achieved, as all post-matching standardized mean differences were ≤0.10, indicating successful matching (Supplementary Figure S1).

In the unmatched cohort, the baseline EASIX values were significantly higher in patients with an adverse clinical course compared with those with a favorable course (median 1.19 vs. 0.91, *p* = 0.008). In contrast, the baseline m-EASIX values were not significantly different between groups (*p* = 0.199). However, the increase in m-EASIX between admission and 48 h (Δm-EASIX) was markedly greater in patients with an adverse clinical course (*p* < 0.001). After propensity score matching (PSM), the primary findings remained consistent. Baseline EASIX values continued to be significantly higher in patients with an adverse clinical course (median 1.25 vs. 0.95, *p* = 0.016), whereas baseline m-EASIX values again did not differ significantly between groups (*p* = 0.183). Notably, Δm-EASIX remained markedly higher in the adverse clinical course group (*p* < 0.001).

In the unmatched cohort, several admission laboratory parameters differed between groups, including higher glucose, BUN, creatinine and LDH levels and lower albumin and corrected calcium levels in patients with an adverse clinical course. After matching, most of these differences were attenuated, indicating an improved baseline comparability between groups; however, LDH levels remained significantly higher in the adverse clinical course group (*p* = 0.002). Inflammatory indices including NLR, PLR, SII, SIRI, and PIV did not significantly differ between groups in either the unmatched or matched cohorts.

### 3.2. Clinical Outcomes and Local Complications

The clinical outcomes and local complications according to clinical course classification in the unmatched cohort are presented in Table 2. As expected from the predefined classification criteria, patients in the favorable clinical course group did not develop local complications, require ICU admission, or experience mortality. Accordingly, patients with an adverse clinical course had a substantially longer hospital stay compared with those with a favorable clinical course. ICU admission occurred in five patients (5.4%) and in-hospital mortality in three patients (3.2%), while pancreatic necrosis and pancreatic abscess were observed in 33 (35.5%) and two (2.2%) patients, respectively. Some patients experienced more than one adverse event.

### 3.3. Predictors of Adverse Clinical Course

In the unmatched cohort, several clinical and laboratory parameters were associated with an adverse clinical course in the univariate analysis, as shown in Table 3. A higher BISAP score (OR 4.395, 95% CI 1.108–17.428, *p* = 0.035) and higher Ranson score (OR 1.875, 95% CI 1.091–3.222, *p* = 0.023) were significantly associated with adverse outcomes. Among endothelial activation indices, both baseline and EASIX (48 h) values were significantly associated with an adverse clinical course (EASIX 0 h: OR 1.323, 95% CI 1.078–1.622, *p* = 0.007; EASIX 48 h: OR 1.588, 95% CI 1.188–2.123, *p* = 0.002). Similarly, m-EASIX values were significantly associated with adverse outcomes (m-EASIX 0 h: OR 1.037, 95% CI 1.009–1.065, *p* = 0.009).

Notably, patients classified as high-risk according to the m-EASIX (48 h) score had a markedly increased likelihood of developing an adverse clinical course (OR 5.541, 95% CI 3.184–9.641, *p* < 0.001). After adjustment for potential confounders, a high m-EASIX (48 h) remained an independent predictor of an adverse clinical course in multivariable analysis (OR 3.056, 95% CI 1.621–5.765, *p* < 0.001). The multivariable logistic regression model demonstrated good calibration according to the Hosmer–Lemeshow goodness-of-fit test (χ^2^ = 7.596, df = 8, *p* = 0.474).

Several inflammatory indices measured at 48 h were also associated with adverse outcomes in the univariate analysis, including NLR (OR 1.186, 95% CI 1.114–1.263, *p* < 0.001), PLR (OR 1.005, 95% CI 1.002–1.008, *p* = 0.003), SII (*p* < 0.001), SIRI (OR 1.172, 95% CI 1.105–1.243, *p* < 0.001) and PIV (*p* < 0.001). However, after multivariable adjustment, only NLR at 48 h remained independently associated with an adverse clinical course (OR 1.106, 95% CI 1.035–1.183, *p* = 0.003).

To confirm the robustness of these findings, the analysis was repeated in the propensity score-matched cohort using cluster-robust logistic regression, as presented in Table 4. In this matched cohort, a high m-EASIX (48 h) remained a significant predictor of an adverse clinical course (univariate OR 1.504, 95% CI 1.275–1.773, *p* < 0.001; multivariable OR 1.355, 95% CI 1.109–1.655, *p* = 0.003). In contrast, the Ranson score was not significantly associated with adverse outcomes in the matched cohort (OR 1.130, 95% CI 0.939–1.360, *p* = 0.194). Among inflammatory markers, NLR (48 h), PLR (48 h), SII (48 h), SIRI (48 h) and PIV (48 h) were significant in univariate analyses but were not retained as independent predictors in multivariable models.

### 3.4. Dynamic Risk Stratification and Predictive Performance

**Figure 2:**
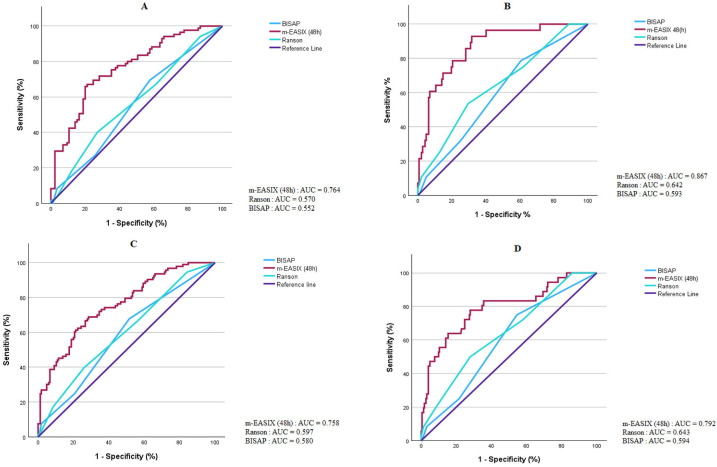
Receiver operating characteristic (ROC) curves comparing the predictive performance of the m-EASIX (48 h), BISAP and Ranson scores for predicting composite and hard outcomes in matched and unmatched cohorts. (**A**) Matched cohort—composite outcome, (**B**) matched cohort—hard outcome, (**C**) unmatched cohort—composite outcome, (**D**) unmatched cohort—hard outcome.

Discrimination analyses in the full unmatched cohort were considered the primary evaluation of predictive performance, whereas the results from the propensity score-matched cohort were interpreted as a robustness analysis. The discriminatory performance of prognostic scoring systems was further evaluated using receiver operating characteristic (ROC) analysis. In the unmatched cohort, the m-EASIX (48 h) score demonstrated a superior predictive performance compared with BISAP and Ranson scores for both composite and hard outcomes, as shown in Table 5 and Figure 2.

**Figure 3:**
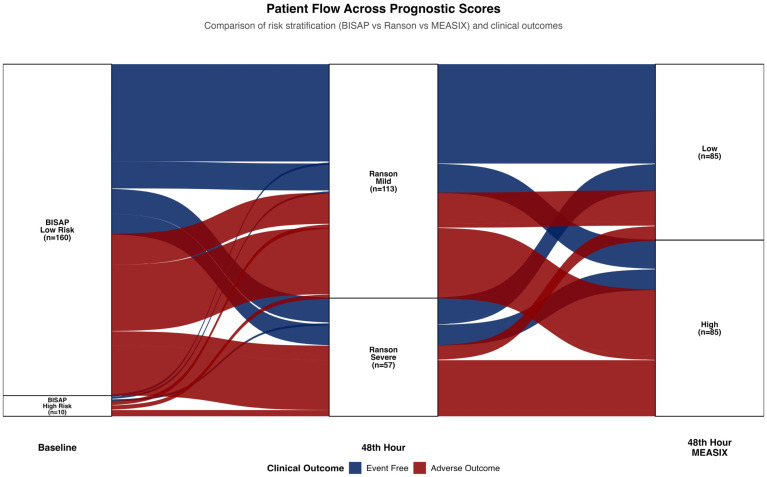
Sankey diagram illustrating longitudinal patient flow across prognostic scoring systems and clinical outcomes. Transitions from baseline BISAP risk categories to 48 h Ranson and m-EASIX (48 h) classifications are shown, highlighting dynamic risk reclassification and its association with favorable and adverse clinical courses.

The Sankey diagram illustrating the longitudinal trajectory of patient risk stratification from baseline to 48 h is presented in Figure 3. Although most of the cohort was initially classified as ‘low-risk’ based on the baseline BISAP score, a re-evaluation at 48 h revealed significant populations at ‘hidden risk.’ Among patients with a low-risk baseline BISAP, the Ranson score reclassified 53 patients as high-risk, 58.5% (31/53) of whom subsequently experienced adverse outcomes. The m-EASIX score demonstrated an even greater sensitivity in detecting latent risk; it reclassified 79 patients from the low-risk baseline to the high-risk category. Notably, 70.9% (56/79) of these patients reclassified by m-EASIX experienced adverse outcomes, compared to only 27.2% (22/81) of those who remained in the low-risk category.

**Figure 4:**
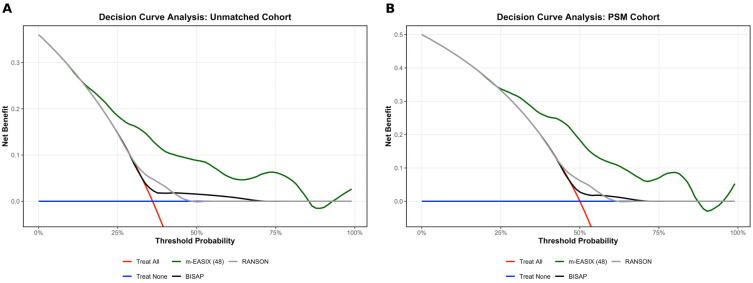
Decision curve analysis comparing the clinical utility of m-EASIX (48 h), BISAP and Ranson scores for predicting adverse clinical outcomes. (**A**) Unmatched cohort; (**B**) matched cohort.

The decision curve analysis (DCA) for the unmatched and matched cohorts is presented in Figure 4. DCA revealed that m-EASIX (48 h) provided a consistently higher net benefit than BISAP and Ranson scores in both the unmatched and propensity score-matched cohorts. Across a wide range of threshold probabilities (approximately 15% to 85%), m-EASIX (48 h) maintained a superior clinical utility, whereas traditional scores reached their functional limits at significantly lower risk thresholds (50–60%). In the unmatched cohort, the addition of m-EASIX (48 h) to BISAP led to marked improvements in risk reclassification and discrimination (NRI = 0.76, 95% CI: 0.52–1.00; IDI = 0.21, 95% CI: 0.16–0.28; *p* < 0.001). Consistent findings were obtained in the matched cohort (NRI = 0.68; IDI = 0.18), indicating a stable model performance across different populations.

## 4. Discussion

In this propensity score-matched cohort, the m-EASIX measured at 48 h was identified as a strong and independent predictor of adverse clinical outcomes in patients with acute pancreatitis (AP). Among several indices based on inflammation and endothelial function, the 48 h m-EASIX showed a higher discriminative performance compared with traditional scoring systems. During the early phase, clinical severity and systemic inflammatory burden may evolve rapidly, making purely admission-based or partially time-dependent scoring systems insufficient for reliable risk classification. In this context, the dynamic reassessment at 48 h enabled the identification of a substantial subgroup of patients with initially low-risk profiles who subsequently developed adverse clinical courses, highlighting the limitations of admission-based risk stratification. Longitudinal flow analysis further illustrates the dynamic transition from low- to high-risk trajectories during the early disease course. By capturing evolving pathophysiological changes, dynamic m-EASIX assessment provides clinically meaningful risk stratification beyond static or semi-dynamic models. A large-scale retrospective study by Wang et al. recently evaluated the prognostic value of EASIX in critically ill patients with AP admitted to the intensive care unit, showing its association with short- and mid-term mortality [26]. While their study focused on admission-based EASIX in a highly selected ICU population, our study expands these findings to a broader cohort of hospitalized patients with AP and emphasizes the additional value of dynamic m-EASIX assessment at 48 h. By using C-reactive protein instead of creatinine, m-EASIX may more accurately reflect the evolving inflammatory burden in non-critically ill patients, thus enhancing its clinical applicability.

The superior predictive performance of m-EASIX at 48 h may be attributed to its strong link with cytokine-driven endothelial dysfunction and microvascular injury in AP [1]. Proinflammatory cytokines, especially interleukin-6 (IL-6), tumor necrosis factor-α (TNF-α) and interleukin-1β (IL-1β), play key roles in enhancing pancreatic inflammation and driving systemic endothelial activation. IL-6 levels usually peak within 24 to 48 h after disease onset and are significantly elevated in severe AP [27,28,29,30]. TNF-α and IL-1β further aggravate endothelial injury by increasing oxidative stress, vascular permeability and leukocyte adhesion, thereby disrupting microvascular integrity and impairing tissue perfusion [31,32]. In addition, chemokines such as CXCL8 (IL-8) and monocyte chemoattractant protein-1 (MCP-1) exacerbate endothelial and microcirculatory injury by recruiting neutrophils and monocytes to inflamed tissues, contributing to vascular barrier damage and the development of local and systemic complications [33,34,35,36]. Together, these inflammatory mediators form a self-amplifying cascade that promotes endothelial dysfunction, microthrombus formation and progression from localized pancreatic inflammation to systemic organ dysfunction.

In this pathophysiological framework, the components of m-EASIX reflect the key downstream consequences of cytokine-mediated endothelial injury. C-reactive protein acts as a primary acute-phase reactant that is largely regulated by IL-6 signaling [28,30]. Elevated levels of CRP measured at 48 h have consistently been associated with severe disease, pancreatic necrosis and adverse clinical outcomes [37]. Various sources of evidence, including clinical studies and guidelines, have shown that CRP values at 48 h are optimal for early risk assessment [37,38,39]. Lactate dehydrogenase (LDH) is a sensitive marker for widespread cellular injury, hypoxia and metabolic stress. Huang et al. found that elevated LDH levels were linked to higher complication rates, increased severity scores and longer hospital stays [40]. Similarly, Cui et al. reported that high LDH levels were an independent predictor of persistent organ failure [41]. Lower platelet counts may suggest an increased activation of the endothelium and consumption due to microthrombi [42,43]. The integration of complementary biological signals allows m-EASIX to serve as a biologically coherent composite marker that reflects both inflammatory burden and cumulative tissue injury.

Importantly, endothelial dysfunction, systemic inflammation and cellular injury develop dynamically during the early stages of AP and usually intensify within the first 48 h [29,31]. The duration of these biological processes is linked to extended hospital stays. Studies have shown that stays in intensive care lasting longer than 7 days are linked to higher mortality rates and greater resource utilization. Additionally, the severity of disease and early spikes in inflammatory markers can predict prolonged hospitalizations [44]. Moreover, implementing structured discharge protocols has demonstrated a reduction in length of stay, emphasizing the significance of early and accurate risk-based clinical decision-making to inform clinical management [45]. In our study, the significantly prolonged hospitalization observed in patients with adverse clinical courses supports the potential role of m-EASIX in reflecting endothelial injury, systemic inflammation and tissue hypoxia, thereby enabling the prediction of both unfavorable outcomes and prolonged hospital stay.

Previous studies have reported that in-hospital mortality rates are approximately 3–4% [24,46], and pancreatic necrosis rates are around 15% among patients with AP [47]. In our cohort, mortality was observed exclusively in the adverse outcome group, whereas the overall necrosis rate was consistent with previously reported data at 15%. Elevated levels of alanine aminotransferase (ALT), particularly those exceeding 1.0 μkat/L, are strongly associated with biliary etiology, exhibiting reported positive predictive values of 80–90% for pancreatitis related to gallstones [48]. In addition, transaminase levels may vary depending on the timing of presentation relative to symptom onset. In the present study, etiology was balanced between groups through propensity score matching. While ALT levels differed between outcome groups in the unmatched cohort and AST levels remained higher in the favorable outcome group after matching, these patterns are most likely related to differences in the timing of presentation rather than underlying etiological imbalance. Multiple studies have shown that inflammation-based indices, such as NLR, PLR and SII, are linked to disease severity and negative outcomes in AP. In a recent single-center cohort study, Salehi et al. found that admission NLR, PLR and SII demonstrated a significant discriminative performance for predicting moderate-to-severe disease [25]. Additionally, a comprehensive systematic review and meta-analysis of 5920 patients confirmed that PLR levels were significantly higher in patients with severe AP compared to those with non-severe cases [49]. In our cohort, several inflammation-based indices were associated with adverse outcomes in univariate analyses. In the unmatched cohort, NLR remained independently associated with adverse outcomes in the multivariable model, whereas this association was not preserved after propensity score matching. Furthermore, inflammatory markers measured at admission showed a limited predictive value for adverse outcomes, highlighting the limitations of relying on a single time-point at presentation.

These findings indicate that, while inflammation-based indices may provide additional insights into outcome assessment, their predictive value appears limited when used alone. Instead, dynamic multi-parameter approaches that capture endothelial injury together with evolving systemic inflammation may enable a more accurate identification of high-risk patients. From a clinical perspective, the identified cut-off value of m-EASIX (48 h) ≥ 15.1 may have practical implications for early risk stratification in patients with acute pancreatitis. Patients exceeding this threshold may represent a subgroup at increased risk for an unfavorable clinical course and could benefit from closer clinical monitoring, earlier imaging evaluation and more intensive supportive management. Conversely, patients with values below this threshold may be more likely to follow a favorable clinical trajectory, which could support more individualized clinical decision-making during the early phase of the disease. Importantly, the m-EASIX score is derived from routinely available laboratory parameters and can therefore be easily implemented in daily clinical practice without additional cost or specialized testing.

This study has several limitations that should be acknowledged. First, its retrospective and single-center design may limit the generalizability of our findings and introduce potential selection bias, even though we employed PSM. In addition, patients with insufficient laboratory data at baseline or at 48 h were excluded because the calculation of the m-EASIX score required these parameters; therefore, selection bias related to missing follow-up laboratory measurements cannot be completely ruled out. Second, while PSM was used to balance key baseline characteristics, we cannot entirely rule out the possibility of residual confounding from unmeasured variables. Third, laboratory parameters were assessed at predefined intervals; however, we could not evaluate more frequent serial measurements beyond 48 h. Fourth, an external validation of the proposed model in independent cohorts and across different clinical centers was not performed; therefore, further studies are required to confirm the generalizability and broader applicability of our findings. Another limitation of this retrospective cohort study is related to the imaging strategy employed. Cross-sectional imaging was conducted based on clinical indications rather than following a standardized imaging protocol. As a result, the choice to perform contrast-enhanced CT or other imaging modalities may have been influenced by the clinical or laboratory parameters evaluated in this study. This could potentially introduce detection bias for complications such as pancreatic necrosis or abscess. Furthermore, patients were not systematically classified based on the revised Atlanta classification due to the limitations inherent in the retrospective dataset. Finally, the relatively low number of mortality events restricted our ability to perform specific analyses related to mortality.

## 5. Conclusions

This propensity score-matched study demonstrates that measuring the modified EASIX (m-EASIX) at 48 h is independently associated with negative clinical outcomes in hospitalized patients with acute pancreatitis. Compared to traditional clinical scores and isolated inflammatory indices, the dynamic assessment of m-EASIX shows a superior discriminative performance by integrating markers of endothelial injury, systemic inflammation, and tissue damage. An m-EASIX threshold of ≥15.1 at 48 h may help distinguish between patients with favorable outcomes and those with adverse clinical courses. These findings emphasize the limitations of relying solely on assessments made at admission and underscore the importance of dynamic reassessment during the early stages of the disease. Due to its simplicity and biological relevance, m-EASIX has the potential to be a useful tool for early risk stratification in acute pancreatitis. Prospective multicenter studies are needed to validate these findings and further clarify the role of m-EASIX in routine clinical practice.

## Figures and Tables

**Table 1:** Baseline demographic, clinical and laboratory characteristics of the unmatched and propensity score-matched cohorts according to clinical course (favorable vs. adverse).

Cohorts	Unmatched			Propensity Score-Matched		
Parameters	Favorable Clinical Course (n = 165)	Adverse Clinical Course (n = 93)	*p*-Value	Favorable Clinical Course (n = 85)	Adverse Clinical Course (n = 85)	*p*-Value
**Age (years), median (IQR)**	54.9 (41.9–72.4)	62.8 (45.0–75.9)	0.089 ^a^	63.1 (45.0–75.2)	63.0 (48.2–76.0)	0.271 ^e^
**Sex, n(%)**						
Male	72 (43.6)	51 (54.8)	0.084 ^b^	50 (58.8)	48 (56.5)	0.850 ^f^
Female	93 (56.4)	42 (45.2)		35 (41.2)	37 (43.5)	
**Etiology, n (%)**						
Biliary	111 (67.3)	62 (66.7)	0.921 ^b^	58 (68.2)	56 (65.9)	0.868 ^f^
Non-biliary	54 (32.7)	31 (33.3)		27 (31.8)	29 (34.1)	
**Clinical scores**						
**BISAP risk group,** **n (%)**						
Low (0–2)	162 (98.2)	86 (92.5)	**0.039 ^c^**	82 (96.5)	78 (91.8)	0.289 ^f^
High (≥3)	3 (1.8)	7 (7.5)		3 (3.5)	7 (8.2)	
**Ranson score (0–48h),** **n (%)**						
Mild (0–2)	122 (73.9)	56 (60.2)	**0.022 ^b^**	62 (72.9)	51 (60.0)	0.100 ^f^
Severe (≥3)	43 (26.1)	37 (39.8)		23 (27.1)	34 (40.0)	
**EASIX score, median (IQR)**	0.91 (0.63–1.30)	1.19 (0.71–1.85)	**0.008 ^a^**	0.95 (0.73–1.50)	1.25 (0.73–1.84)	**0.016 ^e^**
**m-EASIX score, median (IQR)**	1.48 (0.41–5.01)	1.97 (0.45–10.47)	0.199 ^a^	1.75 (0.58–5.65)	2.17 (0.47–11.66)	0.183 ^e^
**Δm-EASIX score, median (IQR)**	5.16 (0.31–12.82)	17.06 (5.52–34.72)	**<0.001 ^a^**	3.44 (0.26–13.89)	17.97 (6.14–35.27)	**<0.001 ^e^**
**NLR**	6.89 (4.11–12.75)	7.34 (4.01–14.62)	0.870 ^a^	8.65 (5.03–14.42)	7.44 (4.06–14.65)	0.343 ^e^
**PLR**	201.8 (125.8–276.9)	187.0 (122.8–303.6)	0.983 ^a^	211.0 (138.2–340.3)	190.7 (123.5–303.3)	0.601 ^e^
**SII**	1733.3 (930.0–3185.5)	2032.3 (935.5–3824.0)	0.385 ^a^	2069.2 (970.3–3576.7)	2032.3 (967.1–3762.0)	0.869 ^e^
**SIRI**	4.01 (2.17–10.23)	4.82 (2.50–9.61)	0.588 ^a^	4.56 (2.71–12.07)	4.85 (2.50–9.75)	0.426 ^e^
**PIV**	989.3 (482.7–2371.6)	1154.3 (561.3–2779.9)	0.225 ^a^	998.4 (605.6–2865.7)	1218.4 (552.7–2700.4)	0.828 ^e^
**Laboratory data (admission)**						
**WBC (10^9^/L), median (IQR)**	11.7 (9.0–15.0)	12.2 (10.2–15.0)	0.144 ^a^	11.4 (9.1–14.8)	12.5 (10.4–14.7)	0.404 ^e^
**Neutrophil (10^9^/L), median (IQR)**	9.3 (6.7–12.5)	9.3 (7.7–13.3)	0.208 ^a^	9.4 (7.1–12.9)	10.1 (7.7–13.1)	0.870 ^e^
**Lymphocyte (10^9^/L), median (IQR)**	1.3 (0.9–1.8)	1.3 (0.8–2.1)	0.597 ^a^	1.10 (0.74–1.64)	1.17 (0.85–2.02)	0.053 ^e^
**Monocyte (10^9^/L), median (IQR)**	0.6 (0.5–0.8)	0.7 (0.5–0.9)	0.181 ^a^	0.7 (0.5–0.8)	0.7 (0.5–0.9)	0.084 ^e^
**Hematocrit (%), median (IQR)**	40.5 (37.4–43.2)	41.2 (36.2–45.5)	0.399 ^a^	40.7 (38.0–43.9)	41.5 (36.2–45.8)	0.650 ^e^
**Platelet (10^9^/L), median (IQR)**	240.0 (189.0–280.5)	240.1 (192.0–302.8)	0.288 ^a^	239.0 (181.0–280.9)	238.0 (190.0–298.0)	0.227 ^e^
**Glucose (mg/dL), median (IQR)**	125 (107–152)	139 (118–169)	**0.015 ^a^**	130 (110–169)	143 (117–171)	0.448 ^e^
**BUN (mg/dL), median (IQR)**	33.0 (22.7–43.5)	39.0 (25.5–50.5)	**0.049 ^a^**	33.0 (25.0–50.0)	39.0 (26.0–51.0)	0.079 ^e^
**Creatinine (mg/dL), median (IQR)**	0.8 (0.7–1.0)	0.9 (0.7–1.2)	**0.042 ^a^**	0.9 (0.7–1.1)	0.9 (0.8–1.2)	0.458 ^e^
**Albumin (g/L), (mean ± SD)**	37.1 ± 4.6	35.6 ± 4.5	**0.016 ^d^**	36.5 ± 4.4	35.7 ± 4.6	0.198 ^g^
**Corrected calcium (mg/dL)**	9.3 (8.9–9.7)	9.1 (8.8–9.5)	**0.043 ^a^**	9.2 (8.9–9.6)	9.1 (8.7–9.5)	0.063 ^e^
**AST (U/L), median (IQR)**	163 (68–319)	128 (46–273)	0.144 ^a^	201 (75–352)	128 (46–271)	**0.033 ^e^**
**ALT (U/L), median (IQR)**	225 (77–391)	136 (49–321)	**0.036 ^a^**	234 (84–371)	136 (50–333)	0.061 ^e^
**Amylase (U/L), median (IQR)**	1520 (725–2372)	1512 (749–2640)	0.471 ^a^	1384 (663–2152)	1501 (815–2509)	0.304 ^e^
**LDH (U/L), median (IQR)**	236 (188–299)	312 (211–412)	**<0.001 ^a^**	241 (195–313)	312 (217–410)	**0.002 ^e^**
**CRP (mg/L), median (IQR)**	1.5 (0.4–5.0)	1.2 (0.3–8.1)	0.578 ^a^	2 (0.5–5.6)	1.5 (0.4–10.3)	0.182 ^e^

^a^: Mann–Whitney U test; ^b^: Pearson chi-square test; ^c^: Fisher’s exact test; ^d^: independent samples *t*-test; ^e^: Wilcoxon signed-rank test; ^f^: McNemar test; ^g^: paired *t*-test; IQR: interquartile range; BISAP: Bedside Index for Severity in Acute Pancreatitis; EASIX: Endothelial Activation and Stress Index; m-EASIX: Modified Endothelial Activation and Stress Index, NLR: Neutrophil–Lymphocyte Ratio; PLR: Platelet–Lymphocyte Ratio; SII: systemic immune inflammation index; SIRI: systemic inflammation response index; PIV: pan-immune inflammation value; WBC: total leukocyte count; BUN: Blood Urea Nitrogen; SD: standard deviation; AST: Aspartate Aminotransferase; ALT: alanine aminotransferase; LDH: lactate dehydrogenase; CRP: C-reactive protein.

**Table 2:** Clinical outcomes and local complications according to clinical course classification in the unmatched cohort.

Parameters	Favorable Clinical Course (n = 165)	Adverse Clinical Course (n = 93)
**Clinical Outcomes**		
Length of stay (days), median (IQR)	4 (3–5)	10 (8–14)
ICU admission, n (%)	0 (0)	5 (5.4)
Mortality, n (%)	0 (0)	3 (3.2)
**Local Complications, n (%)**		
Necrosis	0 (0)	33 (35.5)
Abscess	0 (0)	2 (2.2)

ICU: intensive care unit.

**Table 3:** Analysis of predictors for adverse clinical course in unmatched acute pancreatitis patients using univariate and multivariable logistic regression.

Factor	Univariate Analysis				Multivariate Analysis			
	OR	95% CI		*p*-Value	OR	95% CI		*p*-Value
		Lower	Upper			Lower	Upper	
**Clinical variables**								
Age, years	1.013	0.999	1.026	0.070				
Sex, (male [RC] vs. female)	0.638	0.382	1.063	0.084				
Etiology (biliary [RC] vs. non-biliary)	1.028	0.599	1.764	0.921				
BISAP (0–2 [RC] vs. ≥3)	4.395	1.108	17.428	**0.035**				
Ranson (0–48 h) (0–2 [RC] vs. ≥3)	1.875	1.091	3.222	**0.023**				
**EASIX-based indices**								
EASIX (0 h)	1.323	1.078	1.622	**0.007**				
EASIX (48 h)	1.588	1.188	2.123	**0.002**				
m-EASIX (0 h)	1.037	1.009	1.065	**0.009**				
m-EASIX (48 h) (low [RC] vs. high)	5.541	3.184	9.641	**<0.001**	3.056	1.621	5.765	**<0.001**
**Inflammation-based indices**								
NLR (0 h)	1.004	0.981	1.028	0.730				
NLR (48 h)	1.186	1.114	1.263	**<0.001**	1.106	1.035	1.183	**0.003**
PLR (0 h)	1.001	0.999	1.002	0.389				
PLR (48 h)	1.005	1.002	1.008	**0.003**				
SII (0 h)	1.000	1.000	1.000	0.207				
SII (48 h)	1.001	1.000	1.001	**<0.001**				
SIRI (0 h)	1.008	0.983	1.033	0.550				
SIRI (48 h)	1.172	1.105	1.243	**<0.001**				
PIV (0 h)	1.000	1.000	1.000	0.149				
PIV (48 h)	1.001	1.000	1.001	**<0.001**				

OR: odds ratio, CI: confidence interval, RC: Reference Category, BISAP: Bedside Index for Severity in Acute Pancreatitis, EASIX: Endothelial Activation and Stress Index, m-EASIX: Modified Endothelial Activation and Stress Index, NLR: Neutrophil–Lymphocyte Ratio, PLR: Platelet–Lymphocyte Ratio, SII: systemic immune inflammation index, SIRI: systemic inflammation response index, PIV: pan-immune inflammation value.

**Table 4:** Analysis of predictors of adverse clinical course in propensity score-matched acute pancreatitis patients using univariate and multivariable cluster-robust logistic regression.

Factor	Univariate Analysis				Multivariate Analysis			
	OR	95% CI		*p*-Value	OR	95% CI		*p*-Value
		Lower	Upper			Lower	Upper	
**Clinical variables**								
Ranson (0–48 h) (0–2 [RC] vs. ≥3)	1.130	0.939	1.360	0.194				
**EASIX-based indices**								
EASIX (0 h)	1.026	0.994	1.059	0.108				
EASIX (48 h)	1.041	1.009	1.074	**0.011**				
m-EASIX (0 h)	1.008	1.003	1.014	**0.004**				
m-EASIX (48 h) (Low [RC] vs. High)	1.504	1.275	1.773	**<0.001**	1.355	1.109	1.655	**0.003**
**Inflammation-based indices**								
NLR (0 h)	0.997	0.987	1.008	0.636				
NLR (48 h)	1.336	1.186	1.505	**0.001**				
PLR (0 h)	1.000	0.999	1.001	0.998				
PLR (48 h)	1.001	1.000	1.002	**0.002**				
SII (0 h)	1.000	1.000	1.000	0.967				
SII (48 h)	1.000	1.000	1.000	**<0.001**				
SIRI (0 h)	0.999	0.987	1.011	0.872				
SIRI (48 h)	1.029	1.021	1.037	**<0.001**				
PIV (0 h)	1.000	1.000	1.000	0.742				
PIV (48 h)	1.000	1.000	1.000	**<0.001**				

OR: odds ratio, CI: confidence interval, RC: Reference Category, EASIX: Endothelial Activation and Stress Index, m-EASIX: Modified Endothelial Activation and Stress Index, NLR: Neutrophil–Lymphocyte Ratio, PLR: Platelet–Lymphocyte Ratio, SII: systemic immune inflammation index, SIRI: systemic inflammation response index, PIV: pan-immune inflammation value.

**Table 5:** Comparison of the predictive performance of the m-EASIX (48 h) score with BISAP and Ranson scores for composite and hard outcomes in matched and unmatched cohorts.

Cohort	Outcome	m-EASIX AUC (95% CI)	BISAP AUC (95% CI)	Ranson AUC (95% CI)	DeLong p (m-EASIX vs. BISAP)	DeLong p (m-EASIX vs. Ranson)
PSM (Matched)	Composite	0.764 (0.693–0.835)	0.552 (0.470–0.634)	0.570 (0.487–0.654)	*p* < 0.001	*p* = 0.001
PSM (Matched)	Hard	0.867 (0.799–0.943)	0.593 (0.487–0.699)	0.642 (0.532–0.752)	*p* < 0.001	*p* < 0.001
Unmatched	Composite	0.758 (0.697–0.818)	0.580 (0.513–0.648)	0.597 (0.527–0.666)	*p* < 0.001	*p* < 0.001
Unmatched	Hard	0.792 (0.704–0.881)	0.594 (0.505–0.684)	0.643 (0.550–0.737)	*p* < 0.001	*p* = 0.002

AUC: area under curve, CI: confidence interval, m-EASIX: Modified Endothelial Activation and Stress Index, BISAP: Bedside Index for Severity in Acute Pancreatitis. All ROC analyses were performed using continuous score values. AUC comparisons were conducted using DeLong’s test.

## Data Availability

The datasets used and/or analyzed during the current study are available from the corresponding author upon reasonable request.

## Supplementary Materials

The following supporting information can be downloaded at: https://www.mdpi.com/article/10.3390/medicina62030568/s1, Figure S1: Love plot showing covariate balance before and after propensity score matching. Standardized mean differences (SMDs) for age, sex, BISAP score and etiology are presented, demonstrating adequate balance after matching.
