# Supplementary material for: Dynamic Assessment of Modified EASIX (m-EASIX) at 48 Hours Predicts Adverse Outcomes in Acute Pancreatitis: A Propensity Score-Matched Study
Source: Medicina (Kaunas). 2026 Mar 18;62(3):568. doi: 10.3390/medicina62030568 (PMC13027450; doi:10.3390/medicina62030568)
Supplement: Supplementary file 1 [file medicina-62-00568-s001.zip › medicina-4171693-supplementary.pdf]

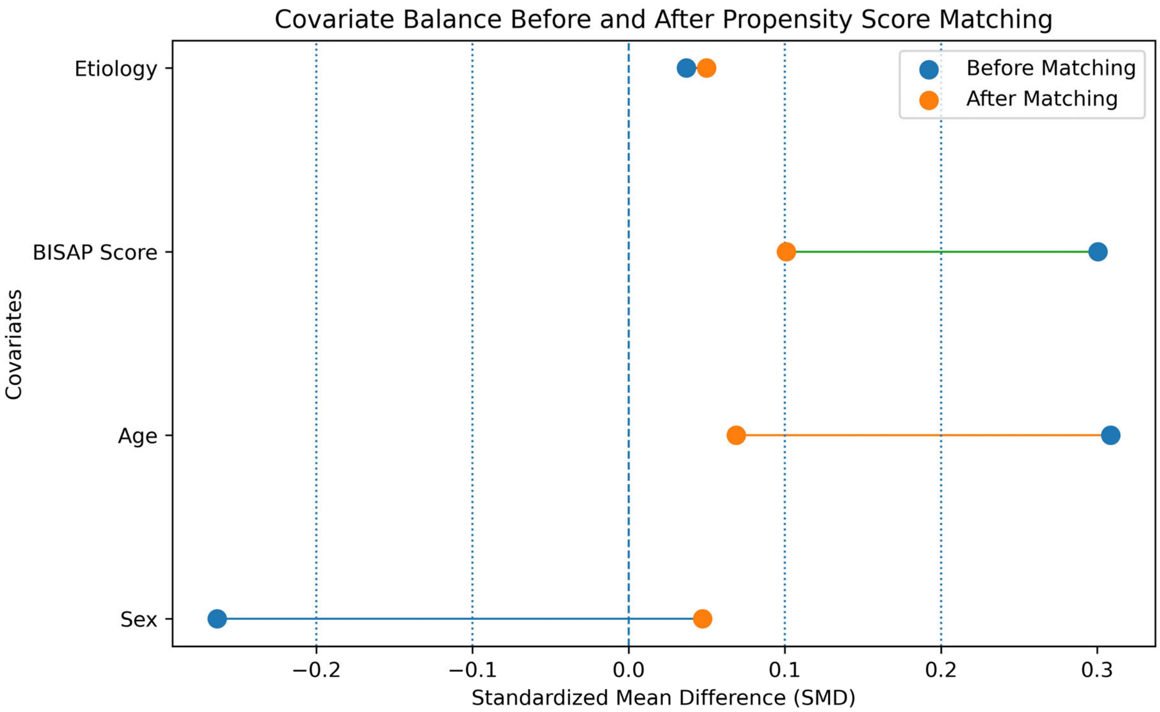

Supplementary Figure S1. Love plot showing covariate balance before and after propensity score matching. Standardized mean differences (SMDs) for age, sex, BISAP score and etiology are presented, demonstrating adequate balance after matching.
